# Genome Characterization of Yellow Fever Virus Wild-Type Strain Asibi, Parent to Live-Attenuated 17D Vaccine, from Three Different Sources

**DOI:** 10.3390/v13071383

**Published:** 2021-07-16

**Authors:** Emily H. Davis, Jill K. Thompson, Steven G. Widen, Alan D. T. Barrett

**Affiliations:** 1 Department of Pathology, University of Texas Medical Branch, Galveston, TX 77555, USA; emidavis@utmb.edu; 2 Department of Biochemistry and Molecular Biology, University of Texas Medical Branch, Galveston, TX 77555, USA; jkthomps@utmb.edu (J.K.T.); sgwiden@utmb.edu (S.G.W.); 3 Sealy Institute for Vaccine Sciences, University of Texas Medical Branch, Galveston, TX 77555, USA

**Keywords:** yellow fever, 17D vaccine, wild-type virus, Asibi, sequencing, consensus compare

## Abstract

The yellow fever virus vaccine, 17D, was derived through the serial passage of the wild-type (WT) strain Asibi virus in mouse and chicken tissue. Since its derivation, the mechanism of attenuation of 17D virus has been investigated using three 17D substrains and WT Asibi virus. Although all three substrains of 17D have been sequenced, only one isolate of Asibi has been examined genetically and all interpretation of attenuation is based on this one isolate. Here, we sequenced the genome of Asibi virus from three different laboratories and show that the WT strain is genetically homogenous at the amino acids that distinguish Asibi from 17D vaccine virus.

## 1. Introduction

Yellow fever virus (YFV) is the causative agent of yellow fever (YF), a hemorrhagic disease involving the liver, kidney and heart. Each year, up to 170,000 cases of YF are reported, with as many as 60,000 related deaths [1]. As there are no antivirals approved for use against YFV, vaccination with the live-attenuated vaccine, termed 17D, is the primary disease control strategy [2]. 

The wild-type (WT) YFV strain Asibi was isolated from the blood of a Ghanaian man with a mild case of YF. The virus was passaged 53 times in rhesus macaque (*Macaca mulatta*) monkeys with periodic passages in *Aedes aegypti* before it was used to derive 17D [3]. The live-attenuated 17D vaccine was derived from the Asibi strain through 176 serial passages in mouse and chicken tissue [2]. Neither the “original” Asibi strain nor the original 17D strain are available today. The 17D vaccine is produced as three substrains, 17D-204, 17D-213 and 17DD, which represent different passages of the original 17D vaccine (i.e., passage 204, 239 and 287–288, respectively, of wild-type strain Asibi). These substrains are considered equally safe and effective as a vaccine.

Many studies have investigated the mechanism of attenuation of 17D virus through the comparison of the vaccine to the Asibi parental strain and have involved genomic sequences of vaccine from different manufacturers of each of the three 17D substrains [3,4]. Thus, there is a rich database of 17D vaccine genomic sequences. In comparison, all the studies on WT Asibi virus are based on the genomic sequence of WT strain Asibi from Yale University; hereafter termed Asibi-Hahn et al. [3,4]. This was a low passage of Asibi virus that had received six passages in Rhesus macaques (*Macaca mulatta*) and three passages in C6/36 cells [5]. Here, we compared the genomic sequences of Asibi virus obtained from three sources—Asibi-Yale (from the Yale Arbovirus Research Unit), Asibi-LSHTM (from the London School of Hygiene and Tropical Medicine) and Asibi-FC (from the Centers of Disease Control and Prevention, Fort Collins, Colorado)—to investigate the homogeneity of Asibi virus obtained from different sources.

## 2. Materials and Methods

Three Asibi viruses from different sources (Yale, LSHTM and FC) and one vaccine ampule of 17D-204 were used in this study. The Asibi-Yale strain was obtained from the World Reference Center for Emerging Viruses and Arbovirus (WRCEVA, Galveston, TX, USA). The virus was received as lyophilized cell culture and was passaged one time in C6/36 cells. The Asibi-LSHTM strain was obtained from the London School of Hygiene and Tropical Medicine [6]. The Asibi-FC strain was obtained from the Centers for Disease Control and Prevention (CDC, Fort Collins, CO, USA) [7]. The 17D-204 vaccine ampule (YF Vax, lot UF795AA, Sanofi Pasteur) was reconstituted according to the manufacturer’s instructions.

Viral RNA was extracted using QiaAmp vRNA kit (Qiagen, Germantown, MD, USA) and sequenced at the UTMB Next Generation Sequencing Core (University of Texas Medical Branch, Galveston, TX, USA). cDNA was constructed using random hexamer primers (TruSeq RNA Library Preparation kit, Illumina, San Diego, CA, USA) and sequenced using the NextSeq550 platform (Illumina, San Diego, CA, USA). Average coverage for Asibi-Yale, Asibi-LSHTM and Asibi-FC was 4689, 964 and 610 reads per base, respectively. Reads were trimmed of adapters and quality filtered for Q-scores greater than 30. De novo consensus sequences were generated using ABySS (v1.3.7) using paired end reads. Full-length sequences can be found on Genbank (Asibi-FC: MT956630, Asibi-LSHTM: MT956629 and Asibi-Yale: MT956628).

## 3. Results

The genomes of Asibi-Yale, Asibi-LSHTM and Asibi-FC were identical in length, 10,862 nucleotides (Genbank accession numbers MT956628, MT956629 and MT956630, respectively). Asibi-Yale and Asibi-FC were very similar, differing by only three nucleotides (nucleotide positions 5509, 6886 and 10800) with no amino acid changes. Asibi-LSHTM is more distinct and differs from the other two Asibi viruses by 17 nucleotides encoding six amino acid substitutions (Table 1). Furthermore, Asibi-LSHTM differs from Asibi-Yale by one additional nucleotide and Asibi-FC by two nucleotides (Table 1). Comparison of West African genotype Asibi-LSTHM-specific amino acid substitutions to wild-type strains from other genotypes revealed that the NS2A-T105A substitution was shared with the equivalent residue in East African, East/Central Africa and Angola genotypes [8].

The Asibi viruses were then compared to all 17D substrains (Table 2). It was shown that the 20 common amino acid residues that differentiate Asibi and 17D are maintained in all Asibi strains. Further, comparison with the only published fatal 17D neurovirulent vaccine revertant (P-16065) showed that none of the amino acid differences between the three Asibi viruses was seen in P-16065 [9].

## 4. Discussion

The Asibi viruses sequenced here differed by varying degrees. Asibi-Yale and Asibi-FC by three, synonymous nucleotides, whereas these two viruses differed from Asibi-LSHTM by 17 nucleotides, resulting in six synonymous changes. All nucleotide changes resulting in amino acid substitutions occurred in the first or second bases of the codon, suggesting that they were a result of selection pressure. The E protein contains three of these amino acid differences (Asibi-Yale and Asibi-FC vs. Asibi-LSHTM) at E-D155R, E-K331R, and E-V412I. Other changes were in the nonstructural proteins: NS1-R10K, NS2A-T105A, and NS4B-V98I. The non-conservative replacement of amino acids at E-155 and NS2A-105 (E-D155R and NS2A-T105A) likely have an impact on protein structure and therefore may impact protein function. E-K331R is an interesting difference between the Asibi strains as all other published genomes of WT YFV strains display an arginine at this position [6,8]. As each 17D substrain also displays an arginine at E-331, this change has not been considered important to the attenuation of 17D and may represent genetic selection during the passage of Asibi-FC and Asibi-Yale (Table 2) [4]. Previous studies have adapted the Asibi-FC strain to hamsters to make it viscerotropic [8,9,10]. Seven hamster passages resulted in seven amino acid substitutions, including E-K331R and NS4B-V98I.

The sequence of the Asibi-Yale virus determined by Hahn et al. (referred to as Asibi-Hahn) is not available on Genbank; however, comparison of Asibi-Hahn et al. to Asibi-Yale revealed 11 synonymous nucleotide changes (nucleotide positions: 2704, 4592, 5362, 5509, 5926, 6013, 6529, 6829, 7496, 7642 7945 and 10243) and four non-synonymous nucleotide changes (Asibi Hahn et al. to Asibi-Yale): E-T153N, NS1-K10R, NS4A-V107I and NS5-D657N) (Table 3). The Asibi-Yale strain used in this study did incur one extra passage in C6/36 cells before it was sequenced, which could account for this nucleotide variation. The E protein of the 17D vaccine substrains is differentially glycosylated with 17DD containing an N-linked glycosylation at E-153-155, 17D-204 virus containing no glycosylation site, and 17D-213 containing an N-linked glycosylation site at E-151-153 (Table 3). Interestingly, none of the Asibi viruses sequenced in this study display an N-linked glycosylation site in E, suggesting that variations in the glycosylation of E do not affect attenuation of 17D in humans [11,12]. Similarly, NS4A-107V and NS5-657N were detected in all Asibi viruses sequenced here and all substrains of 17D, indicating that they likely do not play a role in attenuation. NS1-10 differentiates the Asibi strains with Asibi-LSHTM and all 17D substrains contain NS1-10K, whereas Asibi-FC and Asibi-Yale contain NS1-10R. The impact of this residue on attenuation has not been investigated but, as it not common between all Asibi viruses and 17D substrains, it likely does not play a role in attenuation.

Asibi-Yale has been the most utilized Asibi isolate and was used as the basis of the Asibi infectious clone (i.c.) (Asibi-Yale i.c.) that has been used to define determinants of mosquito competence, genetic diversity and susceptibility to the antiviral Ribavirin [13,14,15,16,17,18,19]. Unsurprisingly, Asibi-Yale i.c. differs from the Asibi Yale used by five synonymous nt changes (Table 2). Previously, Asibi-Yale was the only Asibi virus that had been compared to the 17D vaccine substrains. Even though the Asibi viruses sequenced here were passaged to varying degrees, the 20 common amino acid changes were consistent between the Asibi strains sequenced here and the 17D substrains (Table 2). One difference was noted, the nucleotide change at 3′ untranslated region (3′UTR)-G10,800A, which is common between all Asibi viruses and 17D substrains except for Asibi-FC, and has the vaccine nucleotide (A) at this position. The contribution of this region of the 3′UTR to attenuation has yet to be determined; however, changes to the 3′UTR have been shown to attenuate flaviviruses so it could be important to how Asibi-FC performed in studies [7]. Taken together, although the Asibi strains do differ from each other at a few positions, they do not represent changes that were originally recorded between Asibi and 17D, suggesting that these changes currently identified between WT and vaccine strains can be analyzed with confidence.

## Figures and Tables

**Table 1:** Genomic differences of Asibi strains. Nucleotide (NT) position, gene and amino acid position within protein are recorded. NT changes that do not occur within the coding sequence (CDS) and therefore do not encode an amino acid are recorded as (−).

**NT**	**CDS**	**Asibi-FC**	**Asibi-LSHTM**	**Asibi-Yale**	**Protein #**	**Gene**	**Codon in Gene**	**Asibi-FC**	**Asibi-LSHTM**	**Asibi-Yale**
**887**	769	C	T	C	257	prM	136	L	L	L
**1000**	882	G	A	G	294	E	9	R	R	R
**1437**	1319	A	C	A	440	E	155	D	R	D
**1819**	1701	T	C	T	567	E	282	S	S	S
**1965**	1847	A	G	A	616	E	331	K	R	K
**2207**	2089	G	A	G	697	E	412	V	I	V
**2356**	2238	T	C	T	746	E	461	L	L	L
**2481**	2363	G	A	G	788	NS1	10	R	K	R
**3274**	3156	G	A	G	1052	NS1	274	E	E	E
**3817**	3699	G	A	G	1233	NS2A	103	V	V	V
**3821**	3703	A	G	A	1235	NS2A	105	T	A	T
**3925**	3807	T	A	T	1269	NS2A	139	V	V	V
**4591**	4473	C	T	C	1491	NS3	7	D	D	D
**4864**	4746	G	A	G	1582	NS3	98	Q	Q	Q
**5509**	5391	A	A	G	1797	NS3	313	E	E	E
**6886**	6768	T	C	C	2256	NS4A	149	A	A	A
**7178**	7060	G	A	G	2354	NS4B	98	V	I	V
**7642**	7524	C	T	C	2508	NS5	2	S	S	S
**8917**	8799	C	T	C	2933	NS5	427	V	V	V
**10800**	10682	A	G	G	3561	3’UTR	−	−	−	−

Bolded text indicates NT changes and amino acid substitutions.

**Table 2:** Genomic differences between Asibi strains and the 17D-204 vaccine substrain. Nucleotide (NT) position, gene and amino acid position within protein are recorded. NT changes that do not occur within the CDS and therefore do not encode an amino acid are recorded as (−).

**NT**	**Asibi-FC**	**Asibi- LSHTM**	**Asibi -Yale**	**17D-204**	**17D-213**	**17DD**	**Gene**	**Codon in Gene**	**Asibi-FC**	**Asibi- LSHTM**	**Asibi -Yale**	**17D-204**	**17D-213**	**17DD**
304	G	G	G	A	A	A	C	62	T	T	T	T	T	T
370	T	T	T	C	C	C	C	84	V	V	V	V	V	V
854	C	C	C	T	T	T	prM	125	L	L	L	F	F	F
883	A	A	A	G	G	G	prM	134	T	T	T	T	T	T
887	C	T	C	C	C	C	prM	136	L	L	L	L	L	L
1000	G	A	G	G	G	G	E	9	R	R	R	R	R	R
1127	G	G	G	A	A	A	E	52	G	G	G	R	R	R
1140	C	C	C	T	T	C	E	56	A	A	A	V	V	A
1431	A	A	A	C	C	A	E	153	N	N	N	T	T	N
1437	A	C	A	A	A	T	E	155	D	R	D	D	D	S
1482	C	C	C	T	T	T	E	170	A	A	A	V	V	V
1491	C	C	C	T	T	T	E	173	T	T	T	I	I	I
1572	A	A	A	C	C	C	E	200	K	K	K	T	T	T
1750	C	C	C	T	T	T	E	259	T	T	T	T	T	T
1819	T	C	T	T	T	T	E	282	S	S	S	S	S	S
1870	G	G	G	A	A	A	E	299	M	M	M	I	I	I
1887	C	C	C	T	T	T	E	305	S	S	S	F	F	F
1946	C	C	C	T	T	C	E	325	P	P	P	S	S	P
1965	A	G	A	G	G	G	E	331	K	R	K	R	R	R
2112	A	A	A	G	G	G	E	380	T	T	T	R	R	R
2193	C	C	C	T	T	T	E	407	A	A	A	V	V	V
2219	G	G	G	A	A	G	E	416	A	A	A	T	T	A
2356	T	C	T	T	T	T	E	461	L	L	L	L	L	L
2481	G	A	G	A	A	A	NS1	10	R	K	R	K	K	K
2687	C	C	C	T	C	C	NS1	79	L	L	L	F	L	L
3274	G	A	G	A	A	A	NS1	274	E	E	E	E	E	E
3371	A	A	A	G	G	G	NS1	307	I	I	I	V	V	V
3613	G	A	G	A	A	A	NS2A	35	V	V	V	V	V	V
3622	G	G	G	G	G	G	NS2A	38	L	L	L	L	L	L
3817	G	A	G	G	G	G	NS2A	103	V	V	V	V	V	V
3821	A	G	A	A	A	A	NS2A	105	T	A	T	T	T	T
3860	A	A	A	G	G	G	NS2A	118	M	M	M	V	V	V
3925	T	A	T	T	T	T	NS2A	139	V	V	V	V	V	V
4007	A	A	A	G	G	G	NS2A	167	T	T	T	A	A	A
4013	C	C	C	T	C	C	NS2A	169	L	L	L	F	L	L
4022	A	A	A	G	G	G	NS2A	172	T	T	T	A	A	A
4054	C	C	C	T	T	T	NS2A	182	N	N	N	N	N	N
4056	C	C	C	T	T	T	NS2A	183	S	S	S	F	F	F
4289	A	A	A	C	A	A	NS2B	37	I	I	I	L	I	I
4387	A	A	A	G	G	G	NS2B	69	G	G	G	G	G	G
4505	A	A	A	C	C	C	NS2B	109	I	I	I	L	L	L
4507	T	T	T	C	C	C	NS2B	109	L	L	L	L	L	L
4591	C	T	C	T	T	T	NS3	7	D	D	D	D	D	D
4612	T	T	T	C	C	C	NS3	14	I	I	I	I	I	I
4864	G	A	G	G	G	G	NS3	98	Q	Q	Q	Q	Q	Q
4873	T	T	T	G	G	G	NS3	101	A	A	A	A	A	A
5115	A	A	A	A	G	G	NS3	182	Q	Q	Q	Q	R	R
5153	A	A	A	G	A	A	NS3	195	I	I	I	V	I	I
5194	T	T	T	C	C	C	NS3	208	F	F	F	F	F	F
5362	C	C	C	T	T	T	NS3	264	A	A	A	A	A	A
5431	C	C	C	T	T	T	NS3	287	I	I	I	I	I	I
5473	C	C	C	T	T	T	NS3	301	A	A	A	A	A	A
5509	A	A	G	A	A	A	NS3	313	E	E	E	E	E	E
5926	C	C	C	T	T	T	NS3	452	R	R	R	R	R	R
6023	G	G	G	A	A	A	NS3	485	D	D	D	N	N	N
6448	G	G	G	T	T	T	NS4A	3	A	A	A	A	A	A
6876	T	T	T	C	C	C	NS4A	146	V	V	V	A	A	A
6886	T	C	C	C	C	C	NS4A	149	A	A	A	A	A	A
7171	A	A	A	G	G	G	NS4B	95	I	I	I	M	M	M
7178	G	A	G	G	G	G	NS4B	98	V	I	V	V	V	V
7496	T	T	T	C	C	G	NS4B	204	L	L	L	L	L	S
7571	C	C	C	A	A	A	NS4B	229	R	R	R	R	R	R
7580	T	T	T	C	T	T	NS4B	232	Y	Y	Y	H	Y	Y
7642	C	T	C	C	C	C	NS5	2	S	S	S	S	S	S
7701	A	A	A	G	A	A	NS5	22	Q	Q	Q	R	Q	Q
7945	C	C	C	T	T	T	NS5	103	F	F	F	F	F	F
8008	T	T	T	C	C	C	NS5	124	I	I	I	I	I	I
8629	C	C	C	T	T	T	NS5	331	Y	Y	Y	Y	Y	Y
8808	A	A	A	A	G	G	NS5	391	N	N	N	N	S	S
8917	C	T	C	C	C	C	NS5	427	V	V	V	V	V	V
10142	G	G	G	A	A	A	NS5	836	E	E	E	K	K	K
10285	T	T	T	C	C	C	NS5	883	Y	Y	Y	Y	Y	Y
10312	A	A	A	G	G	G	NS5	892	R	R	R	R	R	R
10338	C	C	C	T	T	T	NS5	901	P	P	P	L	L	L
10367	T	T	T	C	C	C	3’UTR	−	−	−	−	−	−	−
10418	T	T	T	C	C	C	3’UTR	−	−	−	−	−	−	−
10550	T	T	T	C	C	C	3’UTR	−	−	−	−	−	−	−
10800	A	G	G	A	A	A	3’UTR	−	−	−	−	−	−	−
10847	A	A	A	C	C	C	3’UTR	−	−	−	−	−	−	−

Green text indicates Asibi to 17D changes. Genbank accesion numbers used: 17D-204 (KF769015), 17D-213 (U17067.1) and 17DD (U17066.1).

**Table 3:** The Asibi-Yale strain previously sequenced by Hahn et al. was compared to the Asibi-Yale strain sequenced here. Nucleotide (NT) position, gene and amino acid position within protein are recorded. NT changes that do not occur within the CDS and therefore do not encode an amino acid are recorded as (−).

**NT**	**Hahn Asibi-Yale**	**Asibi -Yale**	**17D-204**	**17D-213**	**17DD**	**Gene**	**Codon in Gene**	**Hahn Asibi-Yale**	**Asibi -Yale**	**17D-204**	**17D-213**	**17DD**
304	G	G	A	A	A	C	62	T	T	T	T	T
370	T	T	C	C	C	C	84	V	V	V	V	V
854	C	C	T	T	T	prM	125	L	L	F	F	F
883	A	A	G	G	G	prM	134	T	T	T	T	T
1127	G	G	A	A	A	E	52	G	G	R	R	R
1140	C	C	T	T	C	E	56	A	A	V	V	A
1431	C	A	C	C	A	E	153	T	N	T	T	N
1482	C	C	T	T	T	E	170	A	A	V	V	V
1491	C	C	T	T	T	E	173	T	T	I	I	I
1572	A	A	C	C	C	E	200	K	K	T	T	T
1750	C	C	T	T	T	E	259	T	T	T	T	T
1819	T	T	C	C	C	E	282	S	S	S	S	S
1870	G	G	A	A	A	E	299	M	M	I	I	I
1887	C	C	T	T	T	E	305	S	S	F	F	F
1946	C	C	T	T	C	E	325	P	P	S	S	P
1965	A	A	G	G	G	E	331	K	K	R	R	R
2112	A	A	G	G	G	E	380	T	T	R	R	R
2142	A	C	C	C	C	E	390	H	P	P	P	P
2219	G	G	A	A	G	E	416	A	A	T	T	A
2356	C	T	T	T	T	E	461	L	L	L	L	L
2481	A	G	A	A	A	NS1	10	K	R	K	K	K
2687	C	C	T	C	C	NS1	79	L	L	F	L	L
2704	G	A	A	A	A	NS1	64	V	V	V	V	V
3274	G	G	A	A	A	NS1	274	E	E	E	E	E
3371	A	A	G	G	G	NS1	307	I	I	V	V	V
3613	G	G	A	A	A	NS2A	35	V	V	V	V	V
3860	A	A	G	G	G	NS2A	118	M	M	V	V	V
4007	A	A	G	G	G	NS2A	167	T	T	A	A	A
4013	C	C	T	C	C	NS2A	169	L	L	F	L	L
4022	A	A	G	G	G	NS2A	172	T	T	A	A	A
4054	C	C	T	T	T	NS2A	182	N	N	N	N	N
4056	C	C	T	T	T	NS2A	183	S	S	F	F	F
4289	A	A	C	A	A	NS2B	37	I	I	L	I	I
4387	A	A	G	G	G	NS2B	69	G	G	G	G	G
4505	A	A	C	C	C	NS2B	109	I	I	L	L	L
4507	T	T	C	C	C	NS2B	109	L	L	L	L	L
4591	T	C	T	T	T	NS3	7	D	D	D	D	D
4612	T	T	C	C	C	NS3	14	I	I	I	I	I
4864	A	A	G	G	G	NS3	98	Q	Q	Q	Q	Q
4873	T	T	G	G	G	NS3	101	A	A	A	A	A
5153	A	A	G	A	A	NS3	195	I	I	V	I	I
5194	T	T	C	C	C	NS3	208	F	F	F	F	F
5362	T	C	T	T	T	NS3	264	A	A	A	A	A
5431	C	C	T	T	T	NS3	287	I	I	I	I	I
5473	C	C	T	T	T	NS3	301	A	A	A	A	A
5509	A	G	A	A	A	NS3	313	E	E	E	E	E
5926	T	C	T	T	T	NS3	452	R	R	R	R	R
6013	T	C	C	C	C	NS3	481	P	P	P	P	P
6023	G	G	A	A	A	NS3	485	D	D	N	N	N
6448	G	G	T	T	T	NS4A	3	A	A	A	A	A
6529	C	T	T	T	T	NS4A	30	F	F	F	F	F
6758	G	A	A	A	A	NS4A	107	V	I	I	I	I
6829	G	T	T	T	T	NS4A	130	D	D	D	D	D
6876	T	T	C	C	C	NS4A	146	V	V	A	A	A
7171	A	A	G	G	G	NS4B	95	I	I	M	M	M
7496	C	T	C	C	G	NS4B	204	L	L	L	L	S
7571	C	C	A	A	A	NS4B	229	R	R	R	R	R
7580	T	T	C	T	T	NS4B	232	Y	Y	H	Y	Y
7642	C	T	T	T	T	NS5	2	S	S	S	S	S
7701	A	A	G	A	A	NS5	22	Q	Q	R	Q	Q
7945	C	C	T	T	T	NS5	103	F	F	F	F	F
8008	T	T	C	C	C	NS5	124	I	I	I	I	I
8629	C	C	T	T	T	NS5	331	Y	Y	Y	Y	Y
9605	G	A	A	A	A	NS5	657	D	N	N	N	N
10142	G	G	A	A	A	NS5	836	E	E	K	K	K
10243	A	G	G	G	G	NS5	869	L	L	L	L	L
10285	T	T	C	C	C	NS5	883	Y	Y	Y	Y	Y
10312	A	A	G	G	G	NS5	892	R	R	R	R	R
10338	C	C	T	T	T	NS5	901	P	P	L	L	L
10367	T	T	C	C	C	3’UTR	−	−	−	−	−	−
10418	T	T	C	C	C	3’UTR	−	−	−	−	−	−
10550	T	T	C	C	C	3’UTR	−	−	−	−	−	−
10800	G	G	A	A	A	3’UTR	−	−	−	−	−	−
10847	C	C	A	A	A	3’UTR	−	−	−	−	−	−

Green text indicates Asibi to 17D changes.

## Data Availability

The genomes of Asibi-Yale, Asibi-LSHTM and Asibi-FC have Genbank accession numbers MT956628, MT956629 and MT956630, respectively.
